# Do we need regional guidelines for breast cancer management in the MENA region? MENA Breast Cancer Guidelines project

**DOI:** 10.3332/ecancer.2017.783

**Published:** 2017-11-30

**Authors:** Reham Fayed, Dina Hamza, Heba Abdallah, Mohamed Kelany, Amira Tahseen, Adel T Aref

**Affiliations:** 1 MedicalSurveys-17 Research Group, Heliopolis, Cairo 1001, PO 11757, Egypt; 2 Egypt Air Hospital, Heliopolis, Cairo 1006, PO 11775, Egypt; 3 Dubai Hospital, Al-Baraha, Dubai, PO 7474, UAE; 4 King Abdullah Medical City, Makkah, PO 57657, KSA; 5 Ain Shams University, Cairo 1033, PO 11566, Egypt; 6 University of Adelaide Medical School, South Australian Health and Medical Research Institute, PO 11060, Adelaide, SA 5001, Australia

**Keywords:** breast cancer, guidelines, regional guidelines, MENA region, MedicalSurveys-17

## Abstract

**Purpose:**

Breast cancer is the most common cancer among females worldwide in general and in the Middle East and the North African region (MENA region) in particular. Management of breast cancer in the MENA region faces a lot of challenges, which include younger age at presentation, aggressive behaviour, lack of national breast screening programmes and lack of reliable data registries as well as socioeconomic factors. These factors make applying the international guidelines for breast cancer management very challenging. The aim of this project is to explore the need for a regional breast cancer guideline as well as to screen the clinical practice of breast cancer management in the MENA region.

**Methodology:**

Three web-based designed surveys were sent to more than 600 oncologists in the MENA region from the period of August 2013 to October 2014. Full descriptive data and information regarding the application of international breast cancer guidelines were collected. The software was using the IP address to prevent duplication of collected data. Descriptive analysis and results were shown as numbers and percentages.

**Results:**

During the period of the survey, 104 oncologists responded, representing around an 11% response rate. The majority of replies came from Egypt (59 responses (59%)), followed by Saudi Arabia (ten responses (9.6%)). Fifty-one per cent of responders had more than ten years of experience, and further 31.7% had 5–10 years of experience. Seventy-four per cent were working in governmental hospitals, which is our target sector. There was a major defect in having a genetic counsel unit (78.8% declared an absence of this service), presence of a national breast screening programme (55.8% declared an absence of this service), performing sentinel lymph node biopsy (43.3% declared an absence of this service). The need for regional guidelines for the management of breast cancer was agreed upon by 90.6% of responders.

**Conclusion:**

There is a clear need to improve the management of breast cancer in the MENA region. Creating a national breast screening programme and a reliable database is essential. A regional guideline is required to establish the best possible management of breast cancer according to the patients and disease specification as well as the regional socioeconomic factors and facilities available. There is also a need to improve clinical research that meets the region’s needs.

## Introduction

Breast cancer is a major healthcare problem worldwide generally and in the Middle East and the North African region (MENA region) specifically. Breast cancer represents around 14% of all new cancer cases in the United States according to the SEER statistics factsheet [1]. According to data from the World Health Organization, the incidence of breast cancer in the countries of the MENA region was placed in the second and third ranking (34–64 cases per year) after the United States, Europe and Australia, where cancer incidence is the highest (> 64 cases per year). But this was not associated with the same rate of mortality, where the highest breast cancer-related deaths were recorded in the MENA region countries [2]. In addition to the high cancer-specific mortality in the MENA region, there is another important fact, which is the relatively younger age at presentation (less than 50 years) in the MENA region (around 50% of cases are younger than 50 years of age) in comparison to only 25% in the developed world [3, 4].

An important problem that interferes with breast cancer management in the MENA region is the lack of accurate breast cancer registry. Although the MENA region has a population of around 300–400 million, there is a significant defect in the cancer registry. Most of the available cancer registry is a hospital-based registry rather than a country-based registry.

Most of the guidelines came from the more developed countries like the United States of America (NCCN, ASCO and ASTRO) and Europe (ESMO, ESTRO and NICE guidelines), where there is a great difference in the facilities and socioeconomic factors as well as the capabilities of the healthcare systems. Much effort has been made in the last ten years to create regional guidelines for breast cancer management in the MENA region. Of these we would like to mention the Cancer Registration Project of the Middle East Cancer Consortium [5] and the NCCN–MENA guidelines [6].

The main defects that met these great efforts were the lack of accurate registry as well as the presence of different healthcare systems sometimes even in the same country. This has led to a defect in the regional guidelines, which did not take into consideration the specific criteria of our patient population as well as the availability of facilities, which sometime are not sufficient to apply the international guidelines in breast cancer management.

## Aim of the MBCG project

The aim of our work is to screen the clinical practice in management of breast cancer in the MENA region and to detect to what extent the international guidelines can be applied. Through a collaborative work between MedicalSurveys-17 Research Group and the European School of Oncology (ESO), we have created the MENA Breast Cancer Guidelines (MBCG) project. Using three web-based surveys, we tried to collect as much data as we can regarding the real clinical practice and the availability of different facilities as well as the obstacles that face the oncologists in the MENA region to apply the best clinical practice. The main objectives of the project were to screen the clinical practice in the MENA region, to detect to what extent the socioeconomic factors can affect the application of the guidelines, to assess the need of specific guidelines for the MENA region and finally to put together recommendations that help to improve breast cancer management in the MENA region

## Methodology

Using the database of the MedicalSurveys-17 Research group (600 Oncologists), three web-based surveys were emailed to each oncologist during the period of the project (from August 2013 until October 2014). The project and the links were posted on social and business networks as well as on oncology-related pages. The surveys were emailed through the ESO email system to the ESO database. The software we used did not allow anyone to reply to any survey more than once for each Internet protocol address to try to decrease bias and duplications. The software used allowed descriptive statistical analysis of the results in the form of numbers and percentage.

## Results

During the period of the project, the links of the three surveys were emailed and posted frequently. Based on the market research data, it is estimated that around 800–900 oncologists (mid-senior and senior) are working in the MENA region.

**Figure 1.**
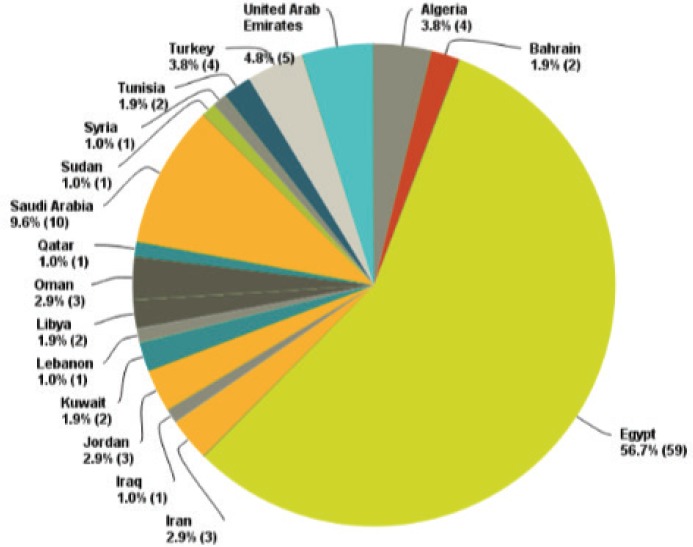
Distribution of countries that participated in the surveys.

We received replies from 104 oncologists with a response rate of around 11%. The replies were distributed among all the countries of the MENA region with a percentage that is proportional to each country’s population and the number of working oncologists in it. Of course, the special situations in Syria and Iraq led to a major defect in getting replies from these countries. The biggest number of replies came from Egypt (56.7%) (Figure 1). This was expected because of the big population (90 million) and the large number of oncologists working at its different institutes.

Of these 104 oncologists, 36.5% were medical oncologists while 32.7% were clinical oncologists, 51% of the oncologists who replied had more than ten years of experience in oncology.

### Type of institute and patient coverage

**Figure 2.**
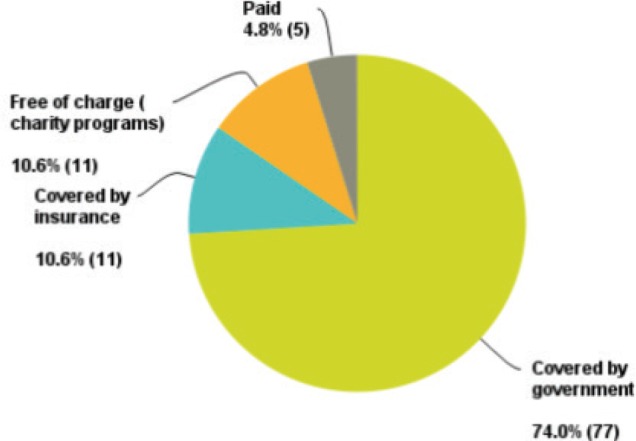
Percentage of replies from the governmental healthcare sector.

Seventy-four per cent of the oncologists who replied have their patient’s treatment covered by the government, which reflects our main target (governmental sector) (Figure 2).

### Patient epidemiology

Sixty-five per cent of the patients at presentation are in the age range of 45–60 years. Sixty-eight per cent of the replies have estimated breast cancer to be representing 50% or more of the total number of cancer cases in each institute. Seventy-seven per cent of the replies stated that more than 50% of cases are presented in advanced or metastatic stage.

### Applying guidelines

**Figure 3.**
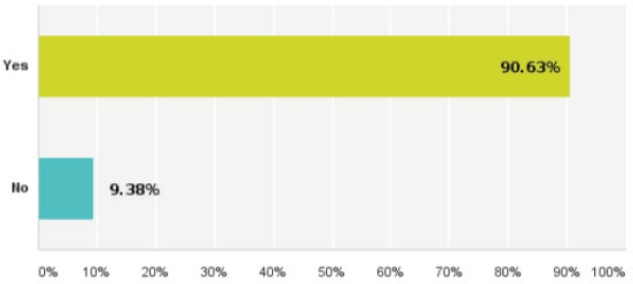
Replies regarding the need for regional guidelines for breast cancer management.

Forty-eight per cent of the replies have chosen the NCCN guidelines to be their institutional guidelines. Meanwhile, 90% have clearly agreed about the ultimate need for regional guidelines (Figure 3).

### Specialised breast cancer unit

Around 65% of the replies reported the presence of a specialised breast cancer unit in their centres; 68.3% have a specialised breast cancer onco-surgeon, but although this percentage seems promising, only 56.7% have sentinel lymph node (SLN) assessment available in their centres. This was mainly due to lack of experience in doing SLN assessment.

Seventy-five per cent of the participants in our project have a centralised pathology laboratory in their centres. An important point to declare is that we did not define the meaning of a centralised pathology laboratory in our surveys; 62.5% have a radio-diagnosis specialist specialised in breast cancer imaging and 68.3% had a radiotherapy facility available in their centre.

### Genetic testing

Only 21.2% of the participants have a genetic counsel unit available in their centres. Around 11.3% have the 21-gene assay available for their patients; 16.9% have it available but not covering all patients; 37.5% do not have any access to 21-gene testing for their patients, while 48.1% have the ability to send the test abroad. However, this is not available for all patients.

### Multidisciplinary approach

About 67.3% of the participants have a multidisciplinary board for breast cancer cases, while 54.8% have the cases discussed initially in the multidisciplinary board.

### Breast cancer screening programme

**Figure 4.**
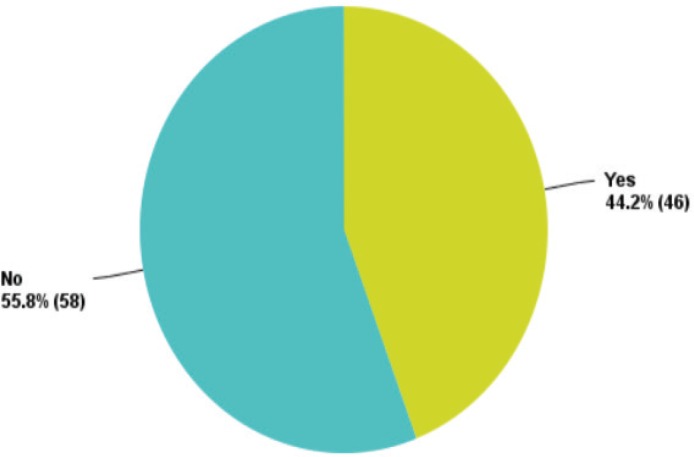
Replies regarding the presence of a national breast cancer screening program.

More than 50% of the participants declared that there is no efficient national or institutional breast cancer screening programme in their country of practice (Figure 4).

### Management and availability of facilities

Our surveys have shown that the essential investigation facilities for screening and diagnosis of breast cancer are acceptably available, with no major defect in the basic essential modalities.

However, there is a tendency to overuse investigative techniques, against the recommendation of the guidelines when the facilities are available. There is a tendency to use PET CT in initial diagnosis of early breast cancer when it is available, as well as the use of bone scan and brain CT and MRI (13% stated that they may use PET CT for all cases if it is available).

### Availability of chemotherapy, targeted therapy and hormonal treatment

Most of the old generation of chemotherapeutic agents were recorded as available in the majority of centres. New generation agents are still not available in a significant percentage of centres (Lapatinib 40%, Eribuline 11% and Ixabeplione 7.7%). New targeted anti-Her-2 agents like Pertuzumab and TDM-1 are not available in the majority of centres (10% and 6.2%, respectively).

## Discussion

The results of our project showed that there is an ultimate need for regional breast cancer guidelines that take into account the specific criteria of breast cancer in the MENA region. It has shown that there is a defect in the availability of national and regional breast screening programmes.

The presence of a specialised breast cancer unit with a specialised onco-surgeon who is an expert in all the advanced surgical techniques in breast cancer, as well as the presence of a specialised radio-diagnosis physician in breast cancer, has become essential for the state-of-the-art care and for the sake of a multidisciplinary approach in breast cancer management [7]. According to the international guidelines, the state of the art in breast cancer management now requires a multidisciplinary team of breast cancer-specialised medical oncologists, radiation oncologists, onco-surgeons, pathologists and radiologists. This is in addition to the availability of specific investigations through which treatment can be tailored according to each patient’s stage including genetic counselling. There is a lot of work needed to fulfil these requirements in the MENA region to be able to cope with the international guidelines.

As mentioned previously, we have a different breast cancer epidemiology in the MENA region compared with that in other parts of the world, with a higher tendency of younger age at presentation and more advanced stages with a higher breast cancer mortality rates. This shows the important need for efficient breast screening programmes to pick up early cases and may also require modifying the age of starting screening and thus creating specific regional criteria for breast screening based on the epidemiological status as well as the available facilities. This is one of the main points that were highlighted by our project. More efficient regional and national efforts are required, which, if achieved, can have a great effect on breast cancer management in terms of decreased treatment cost, as well as improving the breast cancer mortality rates.

Our results represent the governmental (public) sector, which has different, and most of the time less, access to diagnostic and treatment facilities, although it covers the majority of the population in the MENA region. Some facilities may be present in the private sector, but public patients and patients in remote areas may not have access to it. Considering the availability of advanced investigation, medication and targeted therapy, there is a lot of discrepancy between the private and government sectors.

An important point that our results have shown is the tendency to overuse investigations when they are available, out of the guideline’s indications. This may place more burdens on healthcare systems. For successful regional guidelines, there should be a strict indication and a way to control the abuse of the available resources and facilities.

To the best of our knowledge, the MBCG project is the first of its kind to screen the clinical practice in the management of breast cancer in the MENA region. Although different efforts have been made to create specific guidelines for the MENA region, none have considered the differences in patient epidemiology as well the availability or nonavailability of specific facilities that are required either in the investigation or treatment of breast cancer patients. It is clear that modification of the international guidelines is essential to optimise treatment in the MENA region. Furthermore, a different design of clinical trials is also required to address the unique clinical scenarios that arise from the limited resources, which do not occur in more developed countries. This needs the cooperation of all the centres in the MENA region as well as the formation of a breast cancer research network that covers the whole region and is the centre of designing clinical trials.

A clear need for a real and committed breast-screening programme has been shown to be mandatory. This requires the collaboration of all the centres and institutes in the MENA region to create a regional breast screening programme with the aim of decreasing the incidence of advanced breast cancer and breast cancer-related mortality within the next ten years.

Our project has several limitations. The main limitation is the low response rate to our surveys. In addition, there is the inaccuracy of the collected data as well as the difficulty to get a precise response rate because of using online surveys and not face-to-face interaction. Expanding the MedicalSurveys-17 Research Group to include more members from different MENA countries and using a high technology axis allowing face-to-face connection may improve these limitations. The majority of the responses being from Egypt may lead to some bias in results interpretation; however, with our knowledge of the different healthcare systems in the MENA region, we can urge that our results represent a realistic view of clinical practice in the MENA region. We did not cover in detail the distribution of facilities between different countries and also in the same country between major cities and remote cities as well as the difference between the public and private sectors. This may have added more insight into the real practice in the region.

Although the 11% response rate was not enough to establish a solid basis to create regional guidelines, it was enough to highlight the main problems that the oncologists face and the main defects in the clinical practice in the MENA region. These problems, if addressed, could improve clinical practice as well as the level of patient care in the next ten years.

Our impression is that we certainly need a modification of the international guidelines based on our specific patient population and available facilities as well as our socioeconomic factors. This by all means does not mean giving patients suboptimum care, but tailoring our facilities to maximise the care to all patients with no differentiation, and in addition detecting our own problems and trying to solve them through real clinical research that addresses our specific issues.

Our idea of the regional guidelines is that they should address the following points: applying the best possible practice; applying the best alternative when there is a defect in the facilities; collaborative work to make sure that the guidelines are applicable across the whole region,

whatever the healthcare system is; collaborative work to design special clinical trials that address specific problems that may be only present in our region; and, last but not least, revising the guidelines to update them according to the international guidelines, the availability of facilities and the outcome of our trial results.

## Conclusion

There is a clear need to improve the management of breast cancer in the MENA region. Creating a national breast screening programme and a reliable database is essential. Regional guidelines are required to establish the best possible management of breast cancer according to the patients and disease specification as well as the regional socioeconomic factors and available facilities. There is also a need to improve clinical research that meets the region’s needs. Collaborative working between different centres in the region is critical to improve the management of breast cancer in the MENA region.
